# Exploring the Genomic Traits of Non-toxigenic *Vibrio parahaemolyticus* Strains Isolated in Southern Chile

**DOI:** 10.3389/fmicb.2018.00161

**Published:** 2018-02-08

**Authors:** Daniel Castillo, Diliana Pérez-Reytor, Nicolás Plaza, Sebastián Ramírez-Araya, Carlos J. Blondel, Gino Corsini, Roberto Bastías, David E. Loyola, Víctor Jaña, Leonardo Pavez, Katherine García

**Affiliations:** ^1^Marine Biological Section, University of Copenhagen, Helsingør, Denmark; ^2^Instituto de Ciencias Biomédicas, Facultad de Ciencias de la Salud, Universidad Autónoma de Chile, Santiago, Chile; ^3^Departamento de Ciencias Básicas, Facultad de Ciencias, Universidad Santo Tomás, Santiago, Chile; ^4^Laboratory of Microbiology, Institute of Biology, Pontificia Universidad Católica de Valparaíso, Valparaíso, Chile; ^5^I+DEA biotech, Santiago, Chile; ^6^Facultad de Medicina Veterinaria y Agronomía, Universidad de Las Américas, Santiago, Chile; ^7^Departamento de Ciencias Químicas y Biológicas, Universidad Bernardo O′Higgins, Santiago, Chile

**Keywords:** *Vibrio parahaemolyticus*, non-toxigenic, accessory genome, genomic island, prophage, virulence, Zot, RTX

## Abstract

*Vibrio parahaemolyticus* is the leading cause of seafood-borne gastroenteritis worldwide. As reported in other countries, after the rise and fall of the pandemic strain in Chile, other post-pandemic strains have been associated with clinical cases, including strains lacking the major toxins TDH and TRH. Since the presence or absence of *tdh* and *trh* genes has been used for diagnostic purposes and as a proxy of the virulence of *V. parahaemolyticus* isolates, the understanding of virulence in *V. parahaemolyticus* strains lacking toxins is essential to detect these strains present in water and marine products to avoid possible food-borne infection. In this study, we characterized the genome of four environmental and two clinical non-toxigenic strains (*tdh*-, *trh*-, and T3SS2-). Using whole-genome sequencing, phylogenetic, and comparative genome analysis, we identified the core and pan-genome of *V. parahaemolyticus* of strains of southern Chile. The phylogenetic tree based on the core genome showed low genetic diversity but the analysis of the pan-genome revealed that all strains harbored genomic islands carrying diverse virulence and fitness factors or prophage-like elements that encode toxins like Zot and RTX. Interestingly, the three strains carrying Zot-like toxin have a different sequence, although the alignment showed some conserved areas with the *zot* sequence found in *V. cholerae*. In addition, we identified an unexpected diversity in the genetic architecture of the T3SS1 gene cluster and the presence of the T3SS2 gene cluster in a non-pandemic environmental strain. Our study sheds light on the diversity of *V. parahaemolyticus* strains from the southern Pacific which increases our current knowledge regarding the global diversity of this organism.

## Introduction

*Vibrio parahaemolyticus* is a ubiquitous inhabitant in estuaries and marine environments, where it is able to persist and proliferate. This microorganism has been considered an important pathogen causing food-borne illness since the 1950s, because it has the ability to produce major epidemic and pandemic outbreaks worldwide, although only few strains have been proved virulent and most environmental strains are not (Shinoda, 2011). However, it is known that estuaries and marine environments represent a broad reservoir of virulence-associated genes of the genus *Vibrio* (Ceccarelli et al., 2013). These genes may be combined by horizontal gene transfer (HGT) at high frequency and produce pathogenic species if they are incorporated in an appropriate background (Nishibuchi and Kaper, 1995). This is especially important because HGT may lead to emergence of pandemic or pathogenic clones with expanded ecological persistence and dispersion, as occurred in 1996 with the emergence of *V. parahaemolyticus* pandemic strain O3:K6 (Ceccarelli et al., 2013). In Chile, this strain was responsible for large outbreaks of diarrhea and caused thousands of clinical cases until its decline in 2010 (García et al., 2013). However, when the environmental load of the pandemic strain was lower in 2007 and 2009, other non-pandemic but pathogenic clinical strains were able to cause diarrhea, including strains containing virulence genes from the pandemic strain (García et al., 2009). These findings make the emergence of new pathogens from environmental strains a risk to human health (Caburlotto et al., 2010).

The most characteristic virulence-associated factors in *V. parahaemolyticus* are thermostable direct hemolysin (TDH) and the *tdh*-related hemolysin (TRH) (Honda et al., 1988; Nishibuchi et al., 1992; Shinoda, 2011; Zhang and Orth, 2013) which are used to estimate the load of pathogenic strains in seafood during risk analysis. However, diarrhea cases produced by clinical strains lacking *tdh* and *trh* have been reported in many other countries besides Chile (García et al., 2009; Harth et al., 2009; Ottaviani et al., 2012). Virulence studies carried out using environmental strains, including those lacking *tdh/trh*, revealed that these strains have at least some of the virulence characteristics typical of clinical strains; they adhere efficiently to human cells, and once in contact with human intestinal cells they cause disruption of the membrane tight junctions and compromise the intestinal barrier (Mahoney et al., 2010). For this reason, several studies have suggested virulence factors other than TDH and TRH, such as type III and VI secretion systems (T3SS and T6SS, respectively) and pathogenicity islands (VPaI), encoded into the *V. parahaemolyticus* genome (Broberg et al., 2011; Yu et al., 2012; Ceccarelli et al., 2013; Blondel et al., 2016; Hubbard et al., 2016). The presence of additional virulence markers such as genes coding for the apparatus and effectors of the T3SS2 was proposed based on these studies (Yeung et al., 2012). However, Jones et al. (2012) recently reported clinical non-toxigenic strains (negative for *tdh*, *trh*, and T3SS2), indicating that the *tdh*, *trh*, and/or T3SS2 genes are not necessarily predictive of pathogenic potential (Ottaviani et al., 2012). In addition, the genome sequence of *V. parahaemolyticus* RIMD2210633 also revealed the presence of two T6SSs. Notably, while T6SS2 is found in all tested strains of *V. parahaemolyticus*, T6SS1 is found predominantly in clinical isolates (Yu et al., 2012). Both *V. parahaemolyticus* T6SSs were recently proposed to contribute to adhesion to cultured cell monolayers (Salomon et al., 2013), however, most non-pandemic strains had only a partial set of T6SS genes (Ceccarelli et al., 2013). So far, although there have been significant advances in the description of the virulence factors of *V. parahaemolyticus*, the ability of non-toxigenic strains to cause sickness is still not completely understood. Various theories have been proposed to explain why *tdh*-, *trh*-, and T3SS2-negative strains are isolated from sick patients, including coinfection with pathogenic strains, presence of novel and uncharacterized virulence factors and loss of virulence genes during infection (Ronholm et al., 2015). The understanding of virulence in non-toxigenic strains will be essential to detect these strains present in water and marine products and avoid possible food-borne infection.

## Materials and Methods

### Strains and Culture Media

*Vibrio parahaemolyticus* RIMD2210633 (also called VpKX) was obtained from the Research Institute for Microbial Diseases, Osaka University, Osaka, Japan. Chilean clinical strains, identified by the prefix PMC, PMC 53.7 (Harth et al., 2009), and PMC 54.13, were obtained from rectal swabs of patients with acute diarrhea associated with seafood consumption who sought medical attention at the Hospital Regional de Puerto Montt. Diagnosis was confirmed by isolation and identification of *V. parahaemolyticus* in stool cultures and they were sent to the Chilean Institute of Public Health for confirmation according to the Regulation on Notification of Communicable Diseases (Reglamento sobre Notificación de Enfermedades Transmisibles de Declaración Obligatoria N° 712. For details see Figure 3 in Heitmann et al., 2005). VpKX and clinical strains were donated by Professor Romilio Espejo to this study.

Environmental isolate strains, identified by the prefix PMA, were obtained from shellfish samples taken during the summer season (December–February) in Quillaipe, Puerto Montt (**Table 1**). Strains were cultured overnight at 37°C on Luria-Bertani broth (LB), containing 3% NaCl.

**Table 1:** Origin of *V. parahaemolyticus* strains whose DNA sequences were included in this study.

Strain	Origin	Year	Sequencing status	N° contigs	Coverage	N_50_	Accession number
VpKX^a^	Osaka, Japón	1996	Complete	2	Complete	3,288,5888	BA000031.2/BA000032.2
PMC53.7	Puerto Montt, Chile	2007	Draft	27	100x	543,576	MKQF00000000
PMC54.13	Puerto Montt, Chile	2013	Draft	28	113x	509,179	MKQX00000000
PMA14.14	Puerto Montt, Chile	2014	Draft	30	100x	521,587	MKRA00000000
PMA1.15	Puerto Montt, Chile	2015	Draft	130	65x	77,968	MKQV00000000
PMA2.15	Puerto Montt, Chile	2015	Draft	209	60x	42,215	MKQT00000000
PMA3.15	Puerto Montt, Chile	2015	Draft	80	71x	137,938	MKQU00000000


*PMC: Puerto Montt clinical origin; PMA: Puerto Montt environmental origin. ^*a*^*V. parahaemolyticus* strain VpKX has been sequenced previously.*

### Strain Characterization

Samples from clinical cases and shellfish were obtained and analyzed as described previously (Fuenzalida et al., 2006). Briefly, samples of shellfish soft tissue were enriched for *V. parahaemolyticus* in three-tube serial dilutions in alkaline peptone water (APW). Tubes with bacterial growth were tested for *tlh*, *tdh*, and *trh* by multiplex PCR (mPCR) (Bej et al., 1999). Positive *tlh* enrichment tubes were plated on CHROMagar *Vibrio* (CHROMagar Microbiology, Paris, France), and bacterial colonies with the morphology and color expected for *V. parahaemolyticus* were purified. mPCR was performed again using approximately 10 ng of total bacterial DNA per reaction tube; strains positive for *tdh* and/or *trh* were discarded. DGREAs were performed as described previously (Fuenzalida et al., 2006). Information about strains used in this study is showed in **Table 1**.

### Bacterial DNA Extraction

DNA from non-toxigenic strains was extracted from overnight culture of each strain with the Wizard Genomic Purification kit (Promega, Madison, WI, United States). DNA was quantified by UV absorption using a NanoDrop ND-1000 spectrophotometer (Thermo Scientific, Wilmington, DE, United States). Diluted samples of 1 ng/μl DNA in nucleic-free water were used as DNA template for PCR amplification.

### Genome Data Acquisition and Initial Data Processing

DNA sequencing of *V. parahaemolyticus* strains PMA 1.15, PMA 2.15, and PMA 3.15 was performed in Ion Torrent PGM for single-end using 100 bp chemistry libraries (Life Technologies, Carlsbad, CA, United States). *V. parahaemolyticus* strain VpKX was previously sequenced using an Ion Torrent PGM platform (Loyola et al., 2015). DNA sequencing from *V. parahaemolyticus* strains PMC 53.7, PMC 54.13, and PMA 14.14 was performed in Illumina MiSeq platform. Paired-end library preparation and sequencing were performed following the respective manufacturer’s instructions for Illumina TruSeq DNA protocol. For analysis of the sequences, the single-end raw reads from the Ion Torrent were converted into FASTQ format using sff2fastq^1^ software. FASTQ files were analyzed for adapter clipping and quality trimming using Trimmomatic v0.32 (Bolger et al., 2014) with a sliding window of 10, a quality threshold of 15 (Q15), and a minimum sequence length of 35. Reads that passed filters were corrected using POLLUX v1.00 for substitutions, insertions, deletions, and homopolymers (Marinier et al., 2015). All reads that passed all filters were used in downstream analysis. Draft genome assembly was performed using WGS Assembler (Myers et al., 2000) and MIRA (Chevreux et al., 2004) for Illumina MiSeq and Ion Torrent PGM reads, respectively.

### Pan-Genome Analysis

The bioinformatics program EDGAR (Blom et al., 2009) was used to predict the pan-genome (gene repertoire), accessory genome (specific genes, only found in one genome), and core genome (common genes, mutually conserved) of all the *V. parahaemolyticus* strains of this study. Pan-genome development was calculated by iterative pairwise comparison of a set of genomes. Using the metacontigs function of EDGAR, we also defined custom groups of *V. parahaemolyticus* genomes for which the core genome or the pan-genome have been stored as virtual contigs (Blom et al., 2009).

### Phylogenetic Analysis

To determine the phylogenetic relationships among *V. parahaemolyticus* strains based on genomic data, we selected a set of orthologous genes shared by all strains and the outgroup *V. parahaemolyticus* (3,943 genes present in a single copy, paralogs not included) using OrthoMCL with an *e*-value cutoff of 10^-10^ (Chen, 2006). The 3,943 single core genes were first aligned at the amino acid level using Clustal W version 2.0 (Larkin et al., 2007), then back-translated to DNA sequences using PAL2NAL (Suyama et al., 2006). The alignment of all orthologous genes was concatenated using FASconCAT (Kück and Meusemann, 2010). A gene tree was constructed using PhyML (Guindon et al., 2009).

### *In Silico* Identification of the Known *V. parahaemolyticus* Virulence-Associated Genes and Comparative Genomic Analysis of Genomic Islands

Filtered reads were aligned against virulence-related genes described for the pandemic strain of *V. parahaemolyticus* RIMD2210633; the chosen genes were *tdh* (Broberg et al., 2011), MAM7 (Krachler and Orth, 2011), VPaIs (Hurley et al., 2006), T3SS1 including effectors: VopQ, VopS (Yarbrough et al., 2009), VPA0450 (Broberg et al., 2010, 2011), T3SS2 including effectors: VopC, VopT, VopA/P, VopV (Hiyoshi et al., 2011), VopL (Broberg et al., 2011), and genes of both T6SSs (Boyd et al., 2008; Salomon et al., 2013) in all sequenced strain of *V. parahaemolyticus*. Read alignments were performed using SMALT^2^ v0.7.4, with default parameters, producing SAM files; these files were processed using Picard tools v1.96 to convert SAM to BAM and mark duplicate reads. Coverage of virulence genes was calculated by GenomeCoverageBed from the BedTools package (Quinlan and Hall, 2010). Coverage values for all sequences were converted into binary data; 1 for coverage greater than or equal to 75% and 0 for lower coverage values. Finally, the distribution of each gene in every strain was recorded. For comparative analysis, nucleotide sequences were aligned by BLASTN and TBLASTX with the WebACT online resource (Abbott et al., 2007) and visualized with the Artemis Comparison Tool (ACT) release 13.0.0 (Carver et al., 2008).

### Predictions of Genomic Islands, Prophages, and Virulence Factors

We used PAI finder PAIDB v2.0 (Yoon et al., 2015), IslandViewer 4 (Dhillon et al., 2013), and MAUVE v2.3.1 (Darling et al., 2004) to predict the putative genomic islands (GIs) and antimicrobial resistance islands (REIs) in *V. parahaemolyticus* strains. The criterion of selection was based on detection of the GIs by the three tools, presence of mobile-related genes (integrases or transposases) and size > 8 kb. The virulence database MvirDB (Zhou et al., 2007) was used to predict putative virulence factors. All predicted genes of the *V. parahaemolyticus* strains were searched against the MvirDB by BLASTP with loose criteria (*e*-value ≥ 1 × 10^-5^; identity ≥ 35%; coverage ≥ 80%). Also, Virulencefinder 1.2 (Joensen et al., 2014) was used to screen putative virulence factors using selected databases of *Escherichia coli*, *Enterococcus*, and *Streptococcus aureus*. Prophage-like elements were identified by running bacterial genomes in Phage_Finder v2.1 (Fouts, 2006) and PHAST (Zhou et al., 2011).

### Generation of a Phylogenetic Tree Based on Zot Protein Sequences Identified in *Vibrio* Species

To examine the genetic relationship between the Zot proteins in different *Vibrio* species, the amino acid sequences of the Zot proteins in diverse *Vibrio* species were obtained from UniprotKB. Multiple alignments were converted to PHYLIP format using ClustalW 2.0 (Larkin et al., 2007). The phylogenetic reconstruction of sequences was performed using PhyML (Guindon et al., 2009) using the maximum-likelihood method with 100 bootstrap repetitions.

### Examination of the Variability of Zot Proteins in *V. parahaemolyticus* Strains

The variability of Zot proteins in *V. parahaemolyticus* strains was examined by protein alignment using Clustal Omega software (Sievers et al., 2011). The Zot protein of *V. cholera*e O1 El Tor Inaba N16961 (UniProt ID P38442) was used as outgroup.

### Cytotoxicity Assay in Caco-2 Cells

Caco-2 cells were used as the mammalian cell model for the experiment. They were cultured in Dulbecco’s Modified Eagle Medium (DMEM; Gibco^®^, Grand Island, NY, United States) supplemented with 10% fetal bovine serum (FBS; Gibco^®^, Grand Island, NY, United States) plus 1% antibiotic penicillin-streptomycin (Gibco^®^, Grand Island, NY, United States) at 37°C under 5% CO_2_ until confluence. The release of lactate dehydrogenase (LDH) into the medium was measured for the cytotoxicity assay, using the CytoTox96 Non-Radioactive Cytotoxicity Assay kit (Promega, Madison, WI, United States). Briefly, 5 × 10^3^ Caco-2 cells/well were seeded in a curved bottom 96-well plate, using 10% FBS DMEM medium without phenol red or antibiotics (DMEM-LDH test). In parallel, cultures in exponential phase (OD_600_ = 0.6) of the *V. parahaemolyticus* strains tested (listed in **Table 1**) were centrifuged and subsequently a bacterial suspension was prepared in DMEM-LDH test at MOI = 10, then Caco-2 cells were infected with bacteria and incubated for 4 h at 37°C and 5% CO_2_. Finally, LDH release was quantified according to the manufacturer’s instructions, measuring optical density at 490 nm (OD_490_). The percentage of cytotoxicity was calculated with the following equation (Tanabe et al., 2015): ([OD_490_] after experimental release [OD_490_] – after spontaneous release)/([OD_490_] after maximum release [OD_490_] – after spontaneous release) × 100. We performed three independent experiments with three replicates and all analyses were performed in triplicate.

### Statistical Analysis

The values of LDH obtained in the cytotoxicity assay were analyzed with one-way ANOVA using a *post hoc* Bonferroni’s test with 95% significance, using GraphPad Prism 5.0 software. Different cytotoxicity levels were assigned according to statistically significant differences between groups.

## Results

Clinical samples used in this study were obtained from people seeking attention at the Hospital Regional de Puerto Montt after gastrointestinal disease associated with raw mussel ingestion, while environmental strains were obtained from shellfish from Quillaipe, Puerto Montt.

### mPCR and DGREA Classification of *V. parahaemolyticus* Strains

To characterize the clinical and environmental isolates of *V. parahaemolyticus* obtained from Puerto Montt, we performed an initial classification based on the presence/absence of the hemolysin coding genes *tdh*, *trh*, and *tlh* by multiplex PCR and direct genome restriction enzyme analysis (DGREA) pattern. We analyzed 50 strains by mPCR. Most of them (45) were *tdh* and *trh* negative but we chose only five because we discarded samples that we could not perform DGREA analysis (DNA was not cut) and also we discarded clones with equal DGREA patterns. **Table 2** shows a summary of the mPCR and DGREA results. Clinical strains PMC 53.7 (Harth et al., 2009) and PMC 54.13 were classified as *tdh*/*trh* negative and non-pandemic. The remaining four environmental strains (PMA 14.14, 1.15, 2.15, and 3.15) were also *tdh/trh* negative, non-pandemic (**Table 2**) and showed different DGREA patterns (data not shown). The DGREA group of each strain (**Table 2**) was assigned by comparison to previous DGREA patterns obtained for *V. parahaemolyticus* strains that belong to Professor Romilio Espejo. We refer to “pandemic group” to the strains having the same DGREA pattern of the VpKX strain, and “non-pandemic group” to the strains that possess a different DGREA pattern in comparison to VpKX. The comparison of DGREA patterns of the strains analyzed in this work with previous DGREA analysis (García et al., 2013) showed that almost all of them formed new groups (Group 54.13: 2 strains; Group 1.15: 1 strain; Group 2.15: 1 strain, and Group 3.15: 1 strain). PMC53.7 showed the same pattern of strains from group 1.5 previously described (see Figure 3 in García et al., 2013).

**Table 2:** Strain characterization and classification according to mPCR and DGREA.

Strain	mPCR			DGREA (Group)
		
	*trh*	*tdh*	*tlh*	
VpKX	-	+	+	Pandemic (KX) Fuenzalida et al., 2006
PMC 53.7	-	-	+	Non-pandemic (1.5) Harth et al., 2009
PMC 54.13	-	-	+	Non-pandemic (54.13, this study)
PMA 14.14	-	-	+	Non-pandemic (54.13, this study)
PMA 1.15	-	-	+	Non-pandemic (1.15, this study)
PMA 2.15	-	-	+	Non-pandemic (2.15, this study)
PMA 3.15	-	-	+	Non-pandemic (3.15, this study)


### Whole Genome Sequencing and Pan-Genome Analysis of Chilean Isolates

To obtain deeper insight into the genomic diversity of these strains, we performed whole genome sequencing and comparative genomic analysis of each of the environmental and clinical strains isolated from Southern Chile. The genome size of isolates ranged from 4.96 to 5.23 Mb, close to the 5.16 of the VpKX pandemic clone. The G+C content ranged from 45.1 to 45.4%. A total of 4,646–4,991 coding sequences (CDS) were predicted per strain (**Table 3**). To estimate the total gene pool of these *V. parahaemolyticus* strains, we calculated their core and pan-genome with the EDGAR software platform (Blom et al., 2009). These specific analyses defined a conserved “core” genome shared among all strains, interspersed with “accessory” genomic elements that are present in some but absent in other strains. A bacterium contains an open pan-genome when new genes continue to be added to the gene pool of the species any time a new strain is sequenced, whereas a closed pan-genome is designated when the genetic diversity is covered with only a few isolates.

**Table 3:** Genomic overview of the *V. parahaemolyticus* strains analyzed in this study.

Strain	Size (Mbp)	Total genes	Total CDS	%G+C	rRNA	tRNA
VpKX	5.16	4,991	4,831	45.4	34	116
PMC 53.7	5.09	4,649	4,476	45.3	28	98
PMC 54.13	5.13	4,646	4,431	45.3	21	103
PMA 14.14	5.17	4,755	4,586	45.1	25	98
PMA 1.15	5.23	4,843	4,275	45.2	21	75
PMA 2.15	4.96	4,556	4,151	45.4	6	64
PMA 3.15	5.03	4,585	4,316	45.3	4	87


**FIGURE 1:**
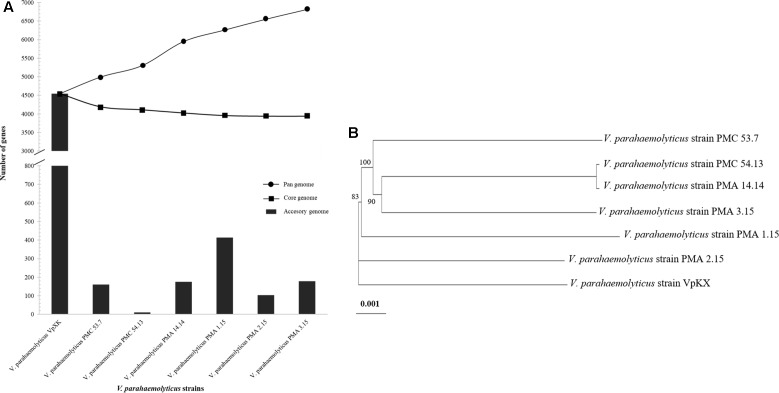
Pan-genome and phylogenetic tree of *V. parahaemolyticus* strains. **(A)** Dynamics of pan-, core, and accessory genes found by progressive addition of *V. parahaemolyticus* genomes. **(B)** Maximum-likelihood tree obtained from a concatenated nucleotide sequence alignment of the orthologous core genes (3,943 genes) for the *V. parahaemolyticus* strains. The VpKX strain was included in the tree. Bootstrap values < 80% are not shown. The horizontal bar at the base of the figure represents 0.001 substitutions per nucleotide site.

Our results showed that the gene repertoire of the *V. parahaemolyticus* pan-genome increased with sequential addition of each new genome, and continued to increase for all additions (6,813 total genes) (**Figure 1A**). In contrast to this increase, an examination of the *V. parahaemolyticus* core genome showed that the number of shared genes decreased with the addition of each new genome (**Figure 1A**). The core genome of these strains was estimated to contain 3,943 genes. To determine the pan-genome of the strains from southern Chile, the accessory genes were calculated. The model estimated that new genes ranged from 11 to 413 for every new *V. parahaemolyticus* sequence added to the analysis (**Figure 1A**). Strains PMA 1.15, PMA 3.15, and PMA 14.14 contained the highest number of accessory genes, which ranged from 160 to 413, while strain PMC 54.13 had only 11 accessory genes. Based on the high rate of increase in the pan-genome and the presence of accessory genes, our data suggest that these specific *V. parahaemolyticus* strains possess an open pan-genome. Interestingly, strains isolated from Puerto Montt had between 148 and 435 fewer genes (annotated open reading frames) than the VpKX pandemic strain (**Table 3**) reflecting the acquisition of diverse genomic islands by the pandemic clone.

Finally, we determined the phylogenetic relationship of these strains using a concatenated alignment of 3943 single-copy orthologs shared by all *V. parahaemolyticus* isolates, including pandemic *V. parahaemolyticus* strain VpKX (**Figure 1B**). The evolutionary tree showed the strains grouped with low genetic diversity. For example, the clinical and environmental *V. parahaemolyticus* strains PMC 54.13 and PMA 14.14, respectively, grouped in the same cluster as we expected according to the DGREA results. *V. parahaemolyticus* strain VpKX was the most distant lineage in the phylogenetic tree (**Figure 1B**).

### Distribution of *V. parahaemolyticus* Virulence-Associated Genes and Genomic Islands

To determine the presence of virulence-related genes previously associated with the pathogenicity of *V. parahaemolyticus*, we performed sequence and comparative genomic analysis to identify the presence of the major virulence factors described for the pandemic strain RIMD2210633 (Hurley et al., 2006; Boyd et al., 2008; Yarbrough et al., 2009; Broberg et al., 2010, 2011; Hiyoshi et al., 2011; Krachler and Orth, 2011; Salomon et al., 2013; Sreelatha et al., 2013). *in Silico* identification of the known pandemic *V. parahaemolyticus* virulence-associated genes and genomic islands (Hurley et al., 2006; Boyd et al., 2008) showed that some, but not all the genes, were identified in some clinical and environmental isolate genomes (Supplementary Table S1). While *tox*R, *tox*S, MAM7, HU2HUalfa, and *tlh* were present in all strains, VPaI-1, VPaI-2, and VPaI-3 were partially present in all strains and some genes that belong to VPaI-4 were found in PMA 2.15. VpaI-6 was almost absent (only PMA 1.15 was positive for two genes of the island), while VpaI-5 and VpaI-7 were absent from all strains.

**FIGURE 2:**
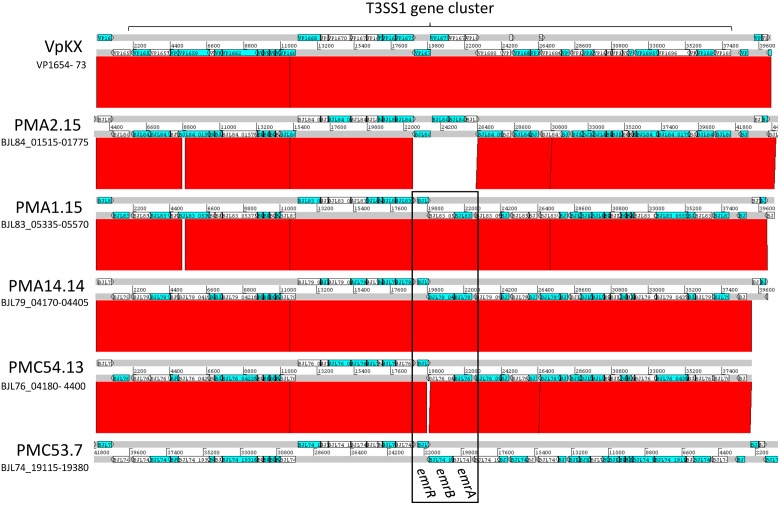
Comparative analysis of the genomic context of the T3SS1 gene cluster in *V. parahaemolyticus* strains. DNA-based comparison of the T3SS1 gene cluster of strains VpKX, PMA2.15, PMA1.15, PMA14.14, PMC54.13, and PMC53.7. The region encoding the putative EmrAB efflux pump and EmrR regulator is highlighted in a box. BlastN analysis was performed using WebACT and displayed with the ACT software.

Comparative genome analysis of the presence and integrity of the T3SS1 and T3SS2 gene clusters among strains from southern Chile showed some interesting differences. Analysis of the T3SS1 gene cluster showed that while strain PMA 2.15 had a genetic structure identical to the pandemic clone, the clinical and environmental strains PMA 1.15, PMA 14.14, PMC 54.13, and PMC 53.7 were missing a set of 4 genes (VP1676–VP1679) in the center of the gene cluster (**Figure 2** and Supplementary Table S1). This set of genes of hypothetical function has not been directly linked to the functionality of the T3SS1, so the impact of their absence in T3SS1 function in these strains is unknown. Interestingly, strains that were missing these genes harbored a module of three new genes in the same position of the cluster, all of which are absent both in the pandemic and PMA 2.15 strains (**Figure 2**). Sequence analysis of the genes encoded in this module predicted functional domains related to the *emrAB* multidrug efflux pump (BJL76_04300 and BJL76_04295 in strain PMC 54.13) and the negative transcriptional regulator of the system *emrR* (BJL76_04290 in PMC 54.13) (**Figure 2**). The presence of this module, within T3SS1 gene clusters, has been recently identified in Acute Hepatopancreatic Necrosis Disease (AHPND)-Causing *V. parahaemolyticus* isolates from China, Thailand, and Mexico (Li et al., 2017). These T3SS1 gene clusters have been renamed T3SS1b to differentiate them from the T3SS1 of *V. parahaemolyticus* RIMD2210633. The presence of this module in non-AHPND strains from southern Chile suggests that T3SS1b is not restricted to AHPND strains. Finally, whether this set of genes encode for a *bona fide* efflux MDR pump and/or contribute to the fitness *V. parahaemolyticus* is yet to be determined.

**FIGURE 3:**
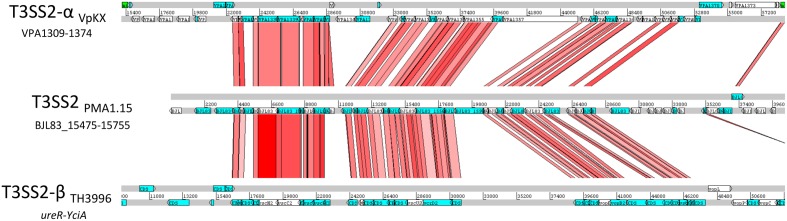
Comparative analysis of the T3SS2 gene clusters identified in strain PMA1.15 of *V. parahaemolyticus*. Translated DNA-based comparison of the T3SS2 gene cluster of PMA1.15 and the T3SS2-α and T3SS2-β of the VpKX and TH3996 strains of *V. parahaemolyticus*, respectively. TBlastX analysis was performed using WebACT and displayed with the ACT software.

The comparative genomic analysis showed absence of VpaI-7, the genomic island which harbors the T3SS2, in each of the *V. parahaemolyticus* strains tested, but further sequence analysis identified the presence of a divergent T3SS2 gene cluster in a different location in the genome of the environmental strain PMA 1.15 (contig 33, accession number NZ_MKQV01000064). Unfortunately, it was not possible to determine the equivalent location in comparison to the *V. parahaemolyticus* RIMD2210633 due to the limited length of contig 33. Comparative genomic analysis of this contig in comparison with the reference strain using Mauve, failed to identify significant homology between the flanking regions of the cluster and the *V. parahaemolyticus* RIMD2210633 genome. In PMA1.15, this cluster encodes for each of the structural components of the system, suggesting that it is functional. Since T3SS2s have been further classified in two major clades (α and β) due mostly to sequence divergence, we performed a comparative analysis of the T3SS2 cluster of PMA1.15 with the T3SS2-α and β systems of the VpKX and TH3996 strains of *V. parahaemolyticus*, respectively (**Figure 3**). The T3SS2 of strain PMA 1.15 had a greater sequence identity and genetic architecture closer to the T3SS2 gene cluster of strain TH3996, so it most likely belongs to the T3SS2-β clade. Interestingly, different studies have identified the presence of T3SS2-β related genes in environmental *tdh-/trh-V. parahaemolyticus* strains (Caburlotto et al., 2009; Paranjpye et al., 2012), suggesting that presence of T3SS2-β in environmental strains might be a global widespread event.

Finally, analysis of the presence and integrity of T6SSs gene clusters in these strains showed that almost all strains except PMA1.15 had T6SS1 (VP1387–VP1414) (Ronholm et al., 2015), although it is associated with clinical more than environmental isolates. T6SS2 was present in all *V. parahaemolyticus* strains (Supplementary Table S1).

### Identification of Novel Genomic Islands in Isolates from Southern Chile

**FIGURE 4:**
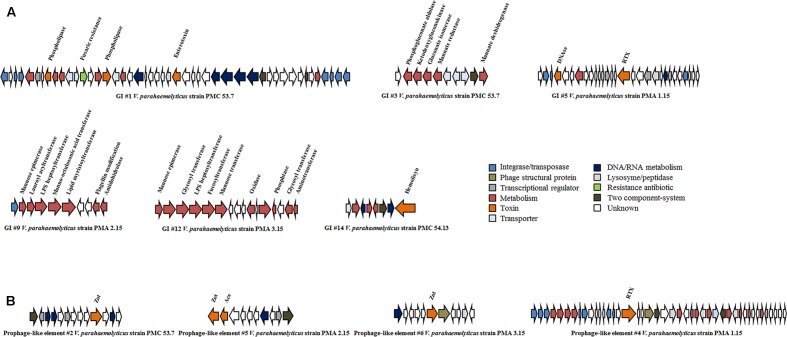
Schematic representation of accessory elements carrying virulence or fitness factors in the *V. parahaemolyticus* strains. **(A)** Genomic islands in *V. parahaemolyticus* strains PMC 53.7, PMC 54.13, PMA 1.15, PMA 2.15, and PMA 3.15. Position of GIs and prophage-like element are shown in **Tables 4**, **5**, respectively. The colors were assigned according to the possible role of each ORF as shown in the figure. **(B)** Prophage-related elements in *V. parahaemolyticus* strains PMC 53.7, PMA 2.15, PMA 3.15, and PMA 1.15 that encode a gene related to *zonula occludens toxin* (*zot*) or RTX toxin.

We further explored the accessory genomes of each *V. parahaemolyticus* isolate to identify novel genes. We detected a total of fifteen strain-specific genomic islands (GIs) between ∼8.0 and 65.1 kb for *V. parahaemolyticus* strains PMC 53.7, PMC 54.13, PMA 14.14, PMA 1.15, PMA 2.15, and PMA 3.15, 7 out of 15 GIs were associated with integrases or transposases (**Table 4**). A total of 393 strain-specific ORFs were found in these GIs and the G+C content ranged from 38.5 to 48.9% (**Table 4**). Genes related to toxins, fitness factors, modification-restriction systems, antibiotic resistance, LPS modification, metabolism, and unknown functions were found in the GIs (**Table 4**). For example, the most cytotoxic strains PMC 53.7, PMA 1.15, and PMA 2.15 presented a diversity of virulence or fitness factors in GIs. *V. parahaemolyticus* strain PMC 53.7 had five specific GIs, one of which had genes that encode phospholipases, enterotoxin, and resistance to the antibiotic fusaric acid (GI #1), a second harbored genes related to gluconate and mannate metabolism (GI #3) (**Figure 4A**). The GI island of strain PMA 1.15 contained DNase and RTX toxin genes (GI #5). GI of strain PMA 2.15 harbored genes related to modification of LPS, including a gene encoding a flagellin modification protein (GI #9) (**Figure 4A**). In addition, the remaining strains were found to encode a diverse group of potential fitness factors. For example, strain PMA 3.15 harbored a GI of 13.6 kb encoding genes related to modification of LPS and O-antigen (GI #12) and the clinical strain PMC 54.13, which harbored only 11 accessory genes, displayed a GI of 8.2 kb that encodes a hemolysin protein (GI #14) (**Figure 4A**). Interestingly, *V. parahaemolyticus* strain PMA 14.14 harbored the longest GI detected (∼171 kb) (**Table 4**). This island harbors 175 genes; we could not identify any potential virulence or fitness factor in this genomic island.

**Table 4:** Features of the 15 strain-specific genomic islands in the *V. parahaemolyticus* genomes.

Strain	GI Number^a^	Contig (position)	Analogous position in VpKX (locus tag)^b^	Length (bp)	ORFs	G + C%	Presence of integrase or transposase	Main feature genes
PMC 53.7	1	1 (681,315–745,872)	VPA1158–VPA1159	64,558	47	45.1	Yes	Phospholipases; antibiotic resistance; enterotoxin
PMC 53.7	2	1 (1,128,115–1,156,441)	VPA0797–VPA0798	28,327	33	40.2	No	Ornithine and sperdimine metabolism
PMC 53.7	3	1 (178,424–189,151)	VPA1709–VPA1711	10,728	10	48.7	No	Glucuronate and mannonate metabolism
PMC 53.7	4	12 (26,197–35,415)	VPA3056–VPA3057	9,219	13	40.1	No	Unknown
PMA 1.15	5	64 (1,686–25,920)	VP2101–VPt050	24,235	29	43.0	Yes	DNAse; RTX toxin
PMA 1.15	6	36 (12,379–27,949)	VP0079–VP0080	15,571	11	41.0	Yes	Type I restriction/modification system
PMA 1.15	7	33 (39,633–50,149)	VPA0113–VPA0114	10,487	5	40.8	Yes	Unknown
PMA 1.15	8	93 (228–10,400)	ND	10,137	11	41.1	No	LPS modification; metabolism
PMA 2.15	9	37 (1–8,835)	ND	8,835	8	39.2	Yes	LPS modification
PMA 2.15	10	4 (103,120–110,414)	VPA0992–VPA0993	8,295	7	40.1	No	CRISPR-associated proteins
PMA 2.15	11	1 (70,850–85,817)	VPAt07–VPA0603	14,968	11	38.5	No	dGTPases
PMA 3.15	12	15 (180,249–193,928)	VPA1167–VPA1168	13,680	17	38.6	Yes	LPS modification
PMA 3.15	13	5 (128,991–137,648)	VPA0391–VPA0392	8,658	8	44.4	No	Transporters; two-component system
PMC 54.13	14	10 (20,491–27,744)	VP1762–VP1763	8,254	8	42.1	No	Hemolysin
PMA 14.14	15	9 (1–170,892)	ND	170,892	175	39.6	Yes	DNA metabolism; unknown


*^*a*^Genomic islands identified simultaneously by PAI finder PAIDB, IslandViewer 4, and MAUVE; ^*b*^ORFs flank the GIs according to strain VpKX genome, annotation; ND: not determined.*

### Identification of Novel Prophages Encoding Putative Zot and RTX Toxins in Isolates from Southern Chile

In addition to the described genomic islands, six different prophage-related elements were detected in the *V. parahaemolyticus* genome sequences; two of these were intact prophages and four defined as incomplete prophages (**Table 5**). Screening of the presence of virulence or fitness factors encoded in these gene clusters showed that *V. parahaemolyticus* strain PMA 1.15 harbored a prophage-like element carrying a putative RTX toxin (**Figure 4B**). In addition, we identified in strains PMC 53.7, PMA 2.15, and PMA 3.15 a prophage-like element of ∼8–11 kb which encoded a putative Zot-like toxin (**Figure 4B**).

**Table 5:** Unique prophage-related sequences distributed in *V. parahaemolyticus* strains.

Prophage	Strain	Size (bp)	#ORFs	%CG	Contig (position bp)	Status
1	PMC 53.7	11,378	13	43.2	6 (179,070–190,447)	Incomplete
2	PMA 1.15	33,500	48	46.1	9 (64,331–97,830)	Complete
3	PMA 1.15	26,568	44	45.8	121 (1–26568)	Complete
4	PMA 2.15	8,380	10	45.3	2 (6,375–14,754)	Incomplete
5	PMA 3.15	10,187	11	45.1	9 (80,993–91,179)	Incomplete


**FIGURE 5:**
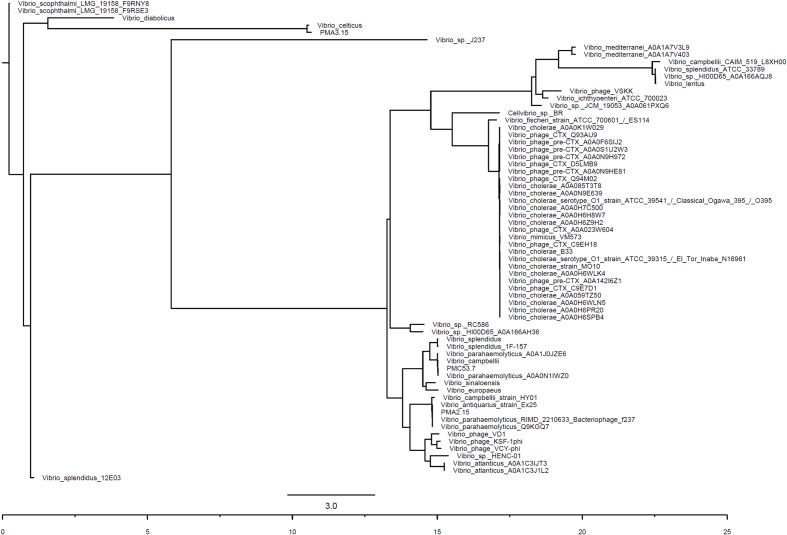
Phylogeny of Zot toxin sequences in *Vibrio* species. Phylogenetic tree based on Zot protein sequences found in diverse *Vibrio* species. Maximum-likelihood method, 100 bootstrap values.

**FIGURE 6:**
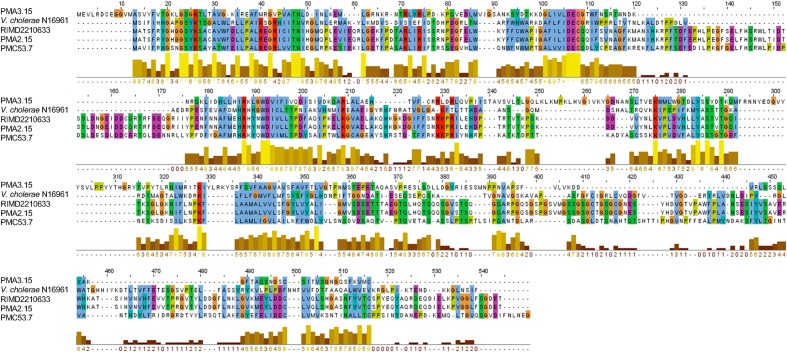
Diversity of Zot toxin sequences in *Vibrio* species. Alignment of Zot amino acid sequences found in *V. parahaemolyticus* strains and *V. cholerae* as outgroup.

Zot toxins have been identified and described in bacterial pathogens such as *V. cholerae* and *Campylobacter spp.*, where they elicit intestinal epithelial barrier damage and cell host death (Di Pierro et al., 2001; Mahendran et al., 2016). In the pandemic VpKX clone, the Zot-like toxin is encoded in the phage f237 (orf7) (Nasu et al., 2000) but its function and role in virulence remains untested. Sequence and phylogenetic analysis showed that only the Zot sequence of PMA 2.15 was identical to that found in the pandemic strain. The rest of the identified Zot proteins from the remainder of the isolates from southern Chile were distinct from the Zot-like toxin of the pandemic strain. Zot from PMC 53.7 was most similar to sequences encoding *zot* in *V. campbellii* and other *V. parahaemolyticus*. The toxin present in PMA 3.15 was the most divergent in sequence and it was grouped with sequences encoding *zot* in *V. celticus* in a further clade (**Figure 5**). Comparison of the sequences using Clustal Omega showed that *V. parahaemolyticus* and *V. cholerae* Zot shared around 24% amino acid identity. Although the clade formed by the coding sequences for Zot present in *V. cholerae* does not include *V. parahaemolyticus* sequences, the alignment analysis of amino acid sequences showed that they share some conserved regions. These regions are located toward the N-terminal domain implied in the morphogenesis of phage more than the C-terminal domain, which is cleaved and secreted into the intestinal lumen in *V. cholerae* (**Figure 6**).

### Evaluation of the Cytotoxic Potential of *V. parahaemolyticus* Strains from Southern Chile

**FIGURE 7:**
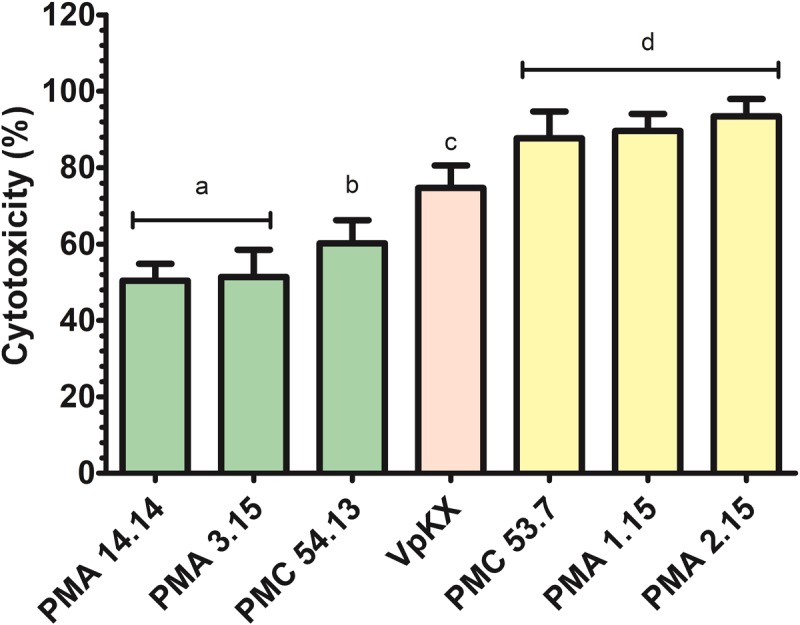
The cytotoxic effect of *V. parahaemolyticus* strains in Caco-2 cells. The release of lactate dehydrogenase (LDH) into the medium was measured by optical density. Different letters indicate as follows: a, medium cytotoxicity; b, medium-high cytotoxicity; c, high cytotoxicity; and d, very high cytotoxicity. ANOVA *post hoc* Bonferroni (*p* < 0.05).

An increasing number of studies have highlighted the isolation of *tdh*-/*trh*-environmental and clinical isolates of *V. parahaemolyticus* with high cytotoxic potential against human intestinal cells grown *in vitro* (Wootipoom et al., 2007; Ottaviani et al., 2012; Thongjun et al., 2013). To test the cytotoxic potential of *V. parahaemolyticus* from southern Chile, we infected human intestinal Caco-2 cells with each strain and determined the levels of LDH release 4 h after infection. As shown in **Figure 7**, the cytotoxicity levels varied among the different *V. parahaemolyticus* strains. We arbitrarily defined three categories for the levels of cytotoxicity observed: medium (20–50%), high (70–90%), and very high (90–100%) levels of cytotoxicity, based on the different groups obtained by statistical analysis. As we expected, the pandemic VpKX produced high cytotoxicity in Caco-2 cells while the clinical *tdh/trh* negative PMC 54.13 showed medium/high cytotoxicity. Interestingly, the environmental strains PMA 1.15, PMA 2.15, and the clinical strain PMC 53.7, all *tdh/trh* negative, caused the most cell damage, surpassing the cytotoxicity displayed by the pandemic strain. Since cytotoxicity in tissue culture cells has been shown to be almost exclusively dependent on T3SS1 (in T3SS2-deficient strains), our results suggest that T3SS1 function is intact in each of the isolates from Puerto Montt and that most likely the gene content differences in the T3SS1 gene cluster that we detected among these strains (**Figure 2**) does not impact the overall capacity of T3SS1 to kill cell culture cells. Additionally, the *V. parahaemolyticus* effector protein VP1680 has been identified as a key factor in the manipulation of signaling and IL-8 secretion in Caco-2 cells (Shimohata et al., 2011). We investigated the phylogenetic relationship of this protein among the seven *V. parahaemolyticus* strains. The tree showed low genetic diversity; however, strains PMC 53.7 and PMA 2.15 displayed very high cytotoxicity and grouped together (Supplementary Figure S1).

## Discussion

The most characteristic virulence-associated factors in *V. parahaemolyticus* are TDH, TRH, and T3SS2 (Honda et al., 1988; Shinoda, 2011; Zhang and Orth, 2013), which have been used as genetic markers for pathogenic strains in seafood during possible food-borne infection (Ceccarelli et al., 2013). However, diarrhea cases produced by clinical non-toxigenic strains lacking *tdh*, *trh*, and T3SS2 have been reported in many countries including Chile (García et al., 2009; Harth et al., 2009; Ottaviani et al., 2012), suggesting that other virulence factors might be contributing to the pathogenicity of *V. parahaemolyticus*.

In this study, we characterized, at the genomic and phenotypic level, a subset of both environmental and clinical strains of *V. parahaemolyticus* isolated in the city of Puerto Montt in southern Chile. Whole-genome sequencing and comparative genomic analysis led to the identification and characterization of a dynamic accessory genome, most likely acquired via lateral gene transfer, which includes genes linked to antibiotic resistance, toxin production, and LPS modification, all of which might improve the fitness of the bacteria (**Figure 1** and **Tables 4**,**5**). Interestingly, similar functions for accessory genes were also found in the *V. parahaemolyticus* pan-genome shown by Ronholm et al., (2015). In contrast, the core genome of these specific *V. parahaemolyticus* strains had 3,943 genes (**Figure 1A**). The size of the core genome was similar to that of a previous study of four clinical *tdh*- and *trh*-negative isolates (Ronholm et al., 2015), but larger than the core genome of the pandemic clone O3:K6 (Chen et al., 2011).

Our analysis highlights the open nature of the pan-genome of these *V. parahaemolyticus* strains (**Figure 1A**), which may be strongly related to the gene gain events occurring in marine environments, where acquisition of new genes is common and allows bacterial communities to have large genomes, a high horizontal rate of gene transfer and several ribosomal operons (Snipen et al., 2009). Genomic islands contribute to the evolution and diversification of microbial communities (Langille et al., 2010). We found 15 GIs that belonged to the accessory genome in all *V. parahaemolyticus* strains analyzed (**Table 4**). Strains PMC 53.7, PMA 1.15, and PMA 2.15 harbored GIs carrying a diversity of virulence factors (**Figure 4A**). For example, strains PMC 53.7 and PMA 1.15 had one GI that encodes phospholipases (GI #1) and RTX toxin (GI #5), which have been associated as key virulence factors contributing to bacterial survival, dissemination, and tissue destruction of the host (Schmiel and Miller, 1999; Welch, 2001; Wiles and Mulvey, 2013; Fiester et al., 2016). Strain PMC 53.7 had a GI (#3) that encodes genes related to gluconate and mannate metabolism, which have been associated with virulence modulation of the pathogenic bacterium *Pectobacterium carotovorum* (Mole et al., 2010) and *V. cholerae* (Roy et al., 2016). Strains PMA 2.15 and PMA 3.15 harbored GIs (#9, #12) that encode genes related to biosynthesis and modification of LPS (**Figure 4A**). The existence of these accessory genes is regarded as an essential virulence factor in *Burkholderia pseudomallei* (Reckseidler et al., 2001), *V. anguillarum* (Castillo et al., 2017), and *V. cholerae* (Chun et al., 2009). More importantly, the presence of these genes has been linked to the modification of capsule polysaccharide content, adherence and evasion of the immune system (Sim et al., 2010). Also, a GI of PMA2.15 carried CRISPR-associated proteins. This is interesting because the outcome of horizontal gene transfer depends on the nature of the incoming DNA and on the genetic background of the host. The CRISPR-Cas systems are able to control the entry of new genetic material by cleaving specific foreign DNA sequences (Samson et al., 2015). Thus, they can clearly interfere with the transfer of exogenous DNA, being a kind of regulator of the acquisition events happening in the environment affecting the bacterial evolution. However, since we did not find complete CRISPR-Cas systems in the *V. parahaemolyticus* genomes, we cannot be sure if they have a role in the genomic evolution of this species.

Finally, clinical strain PMC 54.13 had a unique GI (#14) encoding a hemolysin protein. In *V. cholerae*, this virulence factor causes vacuolation and pore formation in many cell types (Ikigai et al., 1996; Coelho et al., 2000; Mitra et al., 2000) and the purified toxin is rapidly lethal to mice after intravenous administration (Honda and Finkelstein, 1979).

In addition to GIs, prophages also contribute to the evolution of microbial communities. A prophage-like element in strain PMA 1.15 encoded a RTX toxin (**Figure 4B**), which has been associated with bacteria-host interactions and is required for cytotoxic activity in other *Vibrio* species (Chatterjee et al., 2008). Also, the highly cytotoxic strains PMC 53.7, PMA 2.15, and PMA 3.15 harbored a prophage-like element encoding a Zot toxin (**Figure 4B**), which has been described previously in *V. cholerae* as a second toxin (Waldor and Mekalanos, 1996) whose function increases intestinal permeability by interacting with a mammalian cell receptor with subsequent activation of intracellular signaling leading to disassembly of the intercellular tight junctions (Di Pierro et al., 2001; Marinaro et al., 2003). It has also been found in prophage-like elements in *V. coralliilyticus* (Weynberg et al., 2016) and *V. anguillarum* (Castillo et al., 2017), suggesting that the occurrence and exchange of prophages encoding *zot*-like toxins is frequent in *Vibrio* communities. The Zot-like toxin found in strain PMA 2.15 was similar to the toxin encoded in prophage f237 from the pandemic *V. parahaemolyticus* strains belonging to the O3:K6 serovar (Nasu et al., 2000). Interestingly, although we identified three different *zot* sequences in *V. parahaemolyticus*, they shared conserved regions with the *zot* sequence described in *V. cholerae*, suggesting that they could have a similar mechanism of action (**Figure 6**), at least in the function of the N-terminal domain involved in phage morphogenesis. Thus, *V. parahaemolyticus* prophages can potentially enrich the host cell with new genes, which can be highly beneficial for the bacterial host, for example, by enhancing virulence in pathogenic bacteria. Although the role of these toxins in the adaptation and virulence of *V. parahaemolyticus* is unknown and is currently under study in our laboratory, we speculate that the presence of *zot*-encoding prophages in strains PMC 53.7, PMA 2.15, and PMA 3.15 (**Figure 4B**) could have a role in the infection of human intestinal cells, probably causing a major effect of destabilization of the cytoskeleton on the host cells.

It is well established that the dominant contributor to the cytotoxicity is T3SS1 (Hiyoshi et al., 2010). Our results showed that not all the strains shared a common T3SS1 gene cluster architecture, and most of our isolates show absence of the genes of unknown function VP1676–VP1679. Absence of these genes did not affect the cytotoxicity found for each *V. parahaemolyticus* strain, suggesting that they are dispensable for T3SS1-induced killing. In fact, two of the most cytotoxic strains PMA1.15 and PMC53.7 lack all these genes (**Figure 2**). Interestingly, a recent transcriptome analysis of *V. parahaemolyticus* under both T3SS1-inducing conditions and after infection of HeLa cells showed that these genes were not considerably upregulated under either of these conditions, unlike the rest of the T3SS1 gene cluster (Nydam et al., 2014). Together, this suggests that the VP1676-VP1679 genes correspond to a distinct module not directly associated with T3SS1-function.

Subsequent identification of other known virulence factors showed that T6SS1 was present in all strains except PMA1.15 (Supplementary Table S1). This finding was surprising because previous studies demonstrated that gene cluster T6SS1 is more frequently associated with *V. parahaemolyticus* clinical isolates than environmental isolates (Yu et al., 2012) and we did not expect to find this system in almost all analyzed strains. As was previously reported by Ronholm et al., (2015), we also noticed variations in the antibacterial effector VP1388, the immunity protein VP1389 and VP1390 gene (Supplementary Table S1), suggesting that these genes might not be essential to cause cell damage.

Our results show that there is a great genetic diversity in the non-toxigenic *V. parahaemolyticus* strains from southern Chile, including both novel GIs, diversity at the level of T3SS1 gene cluster architecture and the presence of T3SS2-β gene clusters in non-pandemic strains, which add to the current knowledge of the global diversity of this species. Finally, the identification of novel toxin-encoding genes of the RTX and Zot families in different genomic islands and prophages in these strains raises the question of whether they confer a fitness advantage to *V. parahaemolyticus* in both the marine environment and during human infection. Our laboratory is currently working to unravel these interactions and studying if they have a role in the virulence of these strains.

### Accession Numbers

Strains sequenced in this study have the GeneBank accession numbers shown in **Table 1**.

## Author Contributions

KG conceived the study. DC, DP-R, and KG designed and wrote the manuscript. LP and VJ obtained samples and did strain isolation and characterization. NP and SR-A performed the cytotoxicity assay, statistical analysis, and made the figures of the manuscript. GC and RB obtained the DNA samples and prepared libraries for sequencing. CB made the T3SS1 and T3SS2-α/β analysis. DL and KG made the *in silico* identification of known virulence factors. DC performed core and pan-genome analysis and identification of new genomic islands and prophages. DP-R analyzed the *zot* gene diversity. All authors reviewed the manuscript critically and approved the final version.

## Conflict of Interest Statement

The authors declare that the research was conducted in the absence of any commercial or financial relationships that could be construed as a potential conflict of interest.

## Footnotes

1 http://github.com/indraniel/sff2FASTQ

2 http://sanger.ac.uk/resources/software/smalt/
