# Supplementary material for: Exploring the Genomic Traits of Non-toxigenic Vibrio parahaemolyticus Strains Isolated in Southern Chile
Source: Front Microbiol. 2018 Feb 8;9:161. doi: 10.3389/fmicb.2018.00161 (PMC5809470; doi:10.3389/fmicb.2018.00161)
Supplement: Supplementary file 2 [file Image_1.PDF]

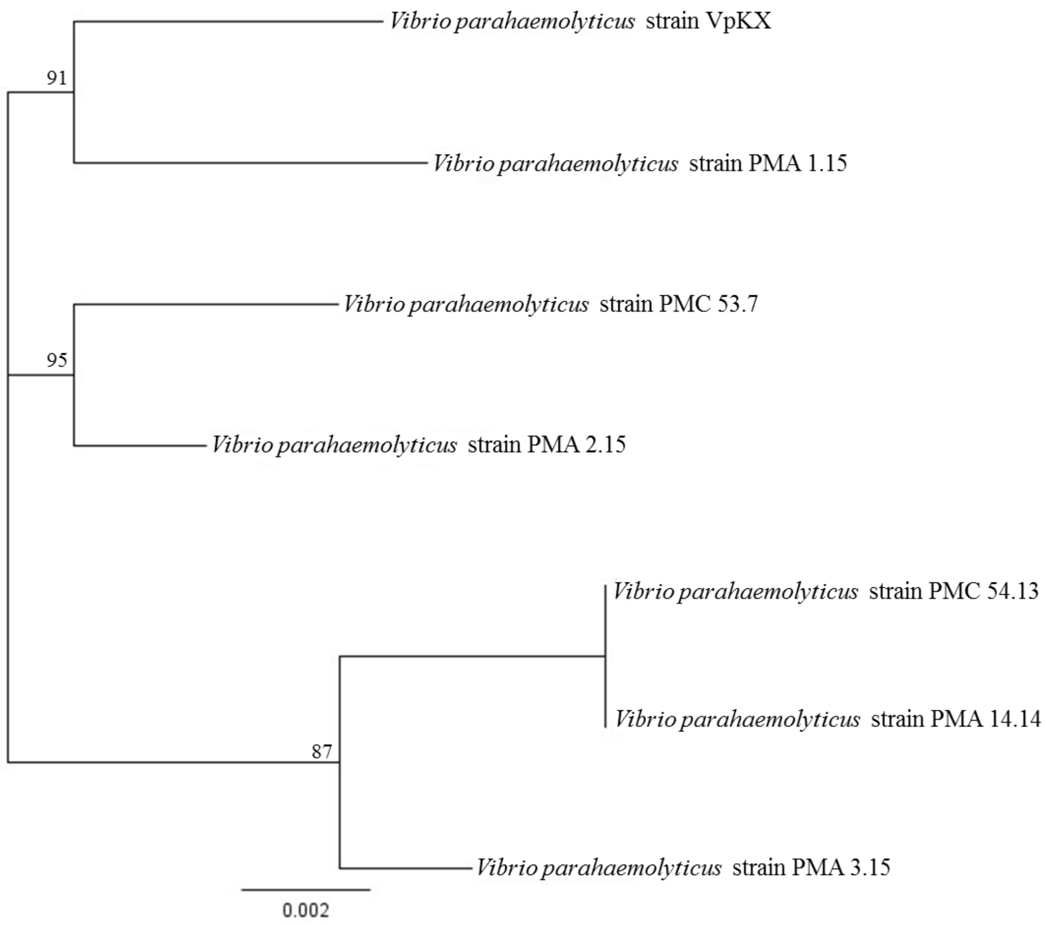

Fig 1S. Phylogenetic tree of VP1680 protein for all *V. parahaemolyticus* strains. The phylogenetic tree was constructed based on the maximum likelihood algorithm, using the amino acid sequence of the protein VP1680 from all the *V. parahaemolyticus* strains analyzed in this study. Bootstrap values >80% were added to the tree. The horizontal bar at the base of the figure represents 0.002 substitutions per amino acid site.
